# Elective Neck Dissection in Patients Undergoing Salvage Laryngectomy: Outcomes, Complications, and Considerations

**DOI:** 10.7759/cureus.60222

**Published:** 2024-05-13

**Authors:** Nickolas A Alsup, Soroush Farsi, Sydney K Blevins, Rachel Giese, Deanne King, Jumin Sunde, Emre Vural, Mauricio Moreno

**Affiliations:** 1 Department of Otolaryngology-Head and Neck Surgery, University of Arkansas for Medical Sciences, Little Rock, USA; 2 Department of Otolaryngology-Head and Neck Surgery, University of Texas Health Science Center at San Antonio, San Antonio, USA

**Keywords:** outcomes, survival, recurrence, occult nodal disease, elective neck dissection, salvage total laryngectomy

## Abstract

Objective

In this study, we sought to identify the predictors for occult nodal disease (OND) and compare oncologic outcomes in patients undergoing elective neck dissection (END) at the time of salvage laryngectomy (SLE) versus the observation group.

Methods

A retrospective chart review was conducted involving all patients with clinically node-negative (cN0) necks who underwent SLE at a tertiary academic center over 12 years. A total of 58 patients met the inclusion criteria and were divided into two groups: END (n=39) and observation (n=19). Primary endpoints were OND, regional recurrence-free survival (RRFS), and disease-specific survival (DSS). Univariate analysis was performed to establish the association between variables with Fisher’s exact test and Mann-Whitney U test. Survival analysis was performed with the log-rank test.

Results

The cohort comprised 46 (79.3%) males and 12 (20.7%) females, with a mean age of 60 years. Pathological nodal disease was identified in five of 71 (7%) examined neck dissection specimens, with positive nodes found in levels II through IV. The only statistically significant predictor of OND was the rT3/rT4 stage (*p*=0.017). There were no differences in perioperative complications, RRFS (*p*=0.216), or DSS (*p*=0.298) between the END and observation groups.

Conclusions

In cN0 necks, the advanced recurrent T-stage (rT3-rT4) is a predictor for OND. As OND was found involving levels II, III, and IV in this study's specimens, formal lateral neck dissection should be the procedure of choice if END is to be performed alongside SLE. While END did not show a significantly higher morbidity profile versus conservative management in this cohort, the procedure did not improve loco-regional control or survival, even when stratifying by tumor stage.

## Introduction

In the setting of advanced laryngeal cancer, salvage laryngectomy (SLE) is generally considered the best available treatment with curative intent for residuals and recurrences, especially for patients without cervical metastasis [1]. There is a wide consensus regarding the role of therapeutic neck dissection for those presenting with node-positive status. Still, whether an elective neck dissection (END) is indicated in the clinically and radiologically node-negative (cN0) patient is unclear. While this procedure is frequently performed as part of routine salvage surgery, a growing body of evidence questions its rationale and oncologic impact.

Among patients undergoing END alone with SLE, the rate of occult nodal disease (OND) varies, reported to range from as low as 0% to as high as 28% [2,3]. Although different strategies have been proposed in the literature, there is no consensus on when END is appropriate [4,5]. Several studies have concluded that END should not be performed at all, in light of the low rates of OND and high morbidity associated with the procedure [6,7].

More studies in the past few years have suggested that at the cohort level, END in itself confers no discernible survival advantage versus observation alone after SLE; individual tumor characteristics such as staging and location along with other considerations such as lifestyle factors and comorbidities need to be assessed on a case-by-case basis to determine whether END is desirable for a particular patient [8-10]. For instance, some studies have identified distinct risk factors for higher rates of OND such as recurrent T3/T4 stage and supraglottic location, concluding that END might only be indicated in the presence of these clinical findings [11,12]. Given the small number of studies on survival rates and the challenges the studies have encountered with regard to meta-analyses, we decided to review our institutional experience, with the objectives of assessing the impact of END on oncologic outcomes, identifying clinicopathological predictors for OND, and establishing a morbidity profile for the procedure [8].

## Materials and methods

This study was conducted at the University of Arkansas for Medical Sciences, a tertiary academic center in Little Rock, Arkansas, USA. The study design received approval from the university's Institutional Review Board (IRB# 228942). Patient selection for the study was based on a retrospective chart review of individuals who had undergone total laryngectomy over 12 years. The inclusion criteria were defined as follows: 1) biopsy-proven recurrent squamous cell carcinoma of the larynx or hypopharynx, including all subsites and stages, 2) prior definitive radiation or concomitant chemoradiation with curative intent, and 3) cN0 neck, based on axial imaging with contrast CT or MRI at the time of re-staging. The exclusion criteria were as follows: 1) primary laryngectomy, 2) partial laryngectomy, 3) clinically or radiologically node-positive neck, 4) functional laryngectomy (aspiration, chondritis), 5) incomplete course of radiation therapy, and 6) elective neck dissection of levels less than II-IV (lateral neck dissection).

Of the 180 potentially eligible patients, 58 were ultimately included in the study, with the most common cause for exclusion being primary laryngectomy. The study group was then divided into END and observation-only cohorts for comparison. Data retrieved from the charts included the following parameters: demographic information, initial and recurrent cancer staging, radiation data, risk factors, tumor characteristics, postoperative course, complications, and oncologic outcomes. Histopathological data included the following aspects: tumor size, margin status, number of lymph nodes, presence and location of pathologically positive nodes, perineural invasion, lymphovascular invasion, and degree of differentiation.

The data analysis was performed using IBM SPSS® Statistics for Windows, Version 22.0. (IBM Corp., Armonk, NY). The primary endpoints were OND, regional recurrence-free survival (RRFS), and disease-specific survival (DSS). Univariate analysis was performed to establish the association between variables with Fisher’s exact test and Mann-Whitney U test. Survival analysis was performed with the log-rank test. A recurrence event was defined as clinical or radiological evidence of disease, with censoring at the last follow-up if no recurrence event occurred by then. For all statistical purposes, significance was set at a *p*-value less than or equal to 0.05.

## Results

Of the 58 patients with a cN0 neck and receiving SLE, 39 (67.2%) underwent END, while 19 (32.8%) had observation only. These two groups were mostly comparable in terms of demographics, tumor staging, and histopathological features, as shown in Table 1. However, a larger proportion of patients completed a chemoradiation course in the END group (*p*=0.013). The study group comprised 46 males and 12 females, with a mean age of 60 years at the time of salvage surgery. The mean radiation dose was 65.7 Gy (range: 51-70.2; SD=4.5), and the average disease-free interval to the diagnosis of the recurrence was 17.8 months. Overall, the mean postoperative follow-up duration for the group was 25 months.

**Table 1:** Demographic and clinicopathological characteristics for the elective neck dissection and observation cohorts ^†^Fisher’s exact test. ^§^Mann-Whitney U test. ^*^Denotes statistical significance END: elective neck dissection

Patient characteristics	END (n=39)	Observation (n=19)	Combined (n=58)	*p*-value
Male, n (%)	29 (74)	17 (89)	46 (79)	0.301^†^
Female, n (%)	10 (26)	2 (11)	12 (21)	0.301^†^
Average time to recurrence, months	16.4	20.6	17.8	n/a
Smoking history, pack years	43	50	45	0.430^§^
Age at laryngectomy, years	59	64	60	0.184^§^
Race, n (%)				
African-American	5 (13)	4 (21)	9 (16)	0.456^†^
White, non-Hispanic	34 (87)	15 (79)	49 (84)	0.456^†^
Tumor site, n (%)				
Supraglottic	17 (44)	5 (26)	22 (38)	0.256^†^
Glottic	9 (23)	9 (47)	18 (31)	0.075^†^
Transglottic	11 (28)	5 (26)	16 (28)	n/a
Hypopharynx	2 (5)	0 (0)	2 (3)	n/a
Recurrent T-stage, n (%)				
rT1	0	2 (11)	2 (3)	0.103^†^
rT2	12 (31)	1 (5)	13 (22)	0.042^†*^
rT3	15 (38)	11 (58)	26 (45)	0.260^†^
rT4	12 (31)	5 (26)	17 (29)	n/a
Histopathological characteristics, n (%)				
Well-moderately differentiated	2 (5)	1 (5)	3 (5)	n/a
Moderately differentiated	25 (64)	11 (58)	36 (62)	0.774^†^
Moderate-poorly differentiated	4 (10)	0	4 (7)	0.292^†^
Poorly differentiated	1 (3)	2 (11)	3 (5)	0.247^†^
Differentiation not specified	0	4 (21)	4 (7)	0.009^†*^
Lymphovascular invasion	8 (21)	3 (16)	11 (19)	n/a
Perineural invasion	13 (33)	5 (26)	18 (31)	0.763^†^
Previous treatment, n (%)				
Radiotherapy	17 (44)	15 (79)	32 (55)	0.013^†*^
Chemoradiotherapy	22 (56)	4 (21)	26 (45)	0.013^†*^

There were only two cases of hypopharyngeal tumors; the vast majority of the patients presented with laryngeal primaries. Among these, glottic, supraglottic, and transglottic locations were similarly represented, as shown in Table 1. On histopathology, extralaryngeal extension was identified in seven cases, and margins were negative (>1 mm) in all but three patients, one of whom had dysplasia at the margin.

Occult nodal disease

From the 39 patients who underwent neck dissection, a total of 71 neck dissection specimens were studied. Only five of these specimens (7%) were found to have microscopic nodal disease, with one patient having bilateral disease. Overall, a total of 1,202 lymph nodes were assessed, and only six of those (0.5%) were microscopically positive for the disease. The clinicopathological characteristics of these patients are summarized in Table 2. The only statistically significant predictor for OND was advanced recurrent T-stage (rT3 and rT4) (*p*=0.017), as shown in Table 3.

**Table 2:** Clinical characteristics of patients with occult nodal disease

Patient	Neck level (# nodes)	Recurrent T-stage	Recurrent location	Initial stage	Initial tumor location	Differentiation
A	IV (1)	T4	Glottic	T1N0	Glottic	Moderate to poor
B	II-IV (2)	T3	Transglottic	T2N0	Glottic	Well
C	II (2)	T3	Supraglottic	T3NX	Supraglottic	Moderate
D	III (1)	T4	Transglottic	Unknown	Transglottic	Well

**Table 3:** Clinicopathological predictors for occult nodal disease ^†^Fisher’s exact test. ^§^Mann-Whitney U test. ^*^Denotes statistical significance

Variable	*p*-value
Age	0.160^§^
Alcohol use (current)	0.162^†^
Smoking status	0.424^†^
Recurrent T-stage	0.017^†*^
Glottic vs. supraglottic	0.768^†^
Positive margins (<1 mm)	0.248^†^

Oncologic outcomes

A comparative analysis of the oncologic outcomes between the groups is presented in Table 4. Overall, at both two years and five years, there were no statistically significant differences in RRFS (*p*=0.216) or DSS (*p*=0.298) between the END and observation groups. Additionally, a subset analysis considering only patients with advanced disease (rT3/rT4) did not show any statistically significant differences in RRFS (*p*=0.942) or DSS (*p*=0.850) between the surgical and observation groups.

**Table 4:** Oncologic outcomes - elective neck dissection vs. observation cohorts ^§^Log-rank test

	Regional recurrence-free survival		Disease-specific survival	
	2-year	5-year	2-year	5-year
Elective neck dissection	77.0%	71.5%	73.5%	63.7%
Observation	93.3%	93.3%	94.1%	78.4%
	*p*=0.216^§^		*p*=0.298^§^	

Postoperative course

Table 5 summarizes the frequency of short- and long-term complications in both groups. The overall pharyngocutaneous (PC) fistula rate in this series was 22.4%, which aligns with the findings in the literature [13]. In the END group, 11 of 39 (28.2%) patients had a fistula postoperatively, while two of 19 (10.5%) presented with this condition in the observation group (*p*=0.186). A subset analysis was performed including only patients with a primary pharyngeal closure. In this subset, six of 23 (26.1%) and two of 10 (20%) had a fistula in the END and observation groups, respectively (*p*=1.000). One patient in the END group died due to myocardial infarction postoperatively. The length of hospital stay was similar in both groups (average of seven days with a median length of stay of 10 days).

**Table 5:** Postoperative complications in the observation and elective neck dissection cohorts ^†^Fisher’s exact test END: elective neck dissection group

Complication	Observation (n=19), n (%)	END (n=39), n (%)	*p*-value
Pharyngocutaneous fistula	2 (10.5)	11 (28.2)	0.186^†^
Pneumonia	2 (10.5)	2 (5.1)	0.446^†^
Cardiovascular	1 (5.3)	3 (7.7)	0.117^†^
Fluid collection	3 (15.8)	3 (7.7)	0.342^†^
Pharyngeal stenosis	1 (5.3)	3 (7.7)	0.732^†^
Required blood transfusion	1 (5.3)	1 (2.6)	0.579^†^

## Discussion

Organ preservation schemes with salvage surgery are the current standard of care for laryngeal cancer in the developed world. While there is little dispute on the need for neck dissection in patients with clinically positive necks, the role of this procedure in cN0 necks has been increasingly challenged. Advances in preoperative imaging continue to push the envelope for identifying low-volume disease in the neck, and such advances likely underlie the high variability in reported OND in surgical specimens [3,14].

In a study examining oral cavity, oropharyngeal, and hypopharyngeal DSS rates, Lee et al. suggested that salvage neck dissection is warranted when recurrence occurs within one year of treatment or if there was initial (pre-radiation) positive nodal disease [15]. Similarly, in a 2021 retrospective study of 171 salvage laryngectomies, Sharma et al. found that nodal positivity before a patient had started initial radiotherapy or chemoradiotherapy predicted occult metastasis on univariable and multivariable analysis [16]. The study suggested that initial nodal positivity warranted offering END to patients later undergoing SLE. Fritz et al. concluded that END improves tumor control in the setting of post-radiation failure and the presence of two or more lymph nodes harboring occult metastatic disease portends a worse prognosis [17]. In a retrospective cohort study, Farlow et al. found that elective paratracheal node dissection at the time of SLE was associated with both improved overall and disease-free survival [12].

A few recent studies have compared survival rates for END versus observation only. A 2020 systematic review and meta-analysis by Gross et al. found no statistically significant difference in overall five-year survival for patients who had END versus observation alone [8]. The primary objective of this 18-study review was to determine rates of OND, and even though the review found 12 studies comparing outcomes between END and observation alone, only three studies could be included in the meta-analysis on survival outcomes. A subsequent cohort study involving 107 patients by Gross et al. found that END accompanying SLE in a cN0 neck was not associated with increased survival versus observation alone after surgery [18]. Two additional systematic reviews appeared in 2019 on this subject [9,10]. The studies agreed that no significant survival advantage had yet been shown in favor of END versus observation-only patients, yet supraglottic recurrence and locally advanced tumors are factors worthy of consideration when deciding whether to proceed with END. Rates of OND are higher in these groups, and END may offer some improved survival benefits in such cases.

In a retrospective review of 125 patients with SLE, Freiser et al. reported no significant association between END and improved survival, though the study did find a significant difference in overall survival in patients with positive versus negative nodal pathology. The study suggested that END may offer prognostic information for approximately 10% of patients and this should be discussed with the patient to decide whether to proceed with END [19]. Similarly, a retrospective study of 23 cases by Gouzos et al. found no significant survival advantage for END versus observation in cN0 necks undergoing SLE, yet END may result in a small percentage of patients being upstaged [20]. Bernard et al., in a retrospective review of 86 patients, found overall survival to be higher (*p*=0.037) for cN0 patients who had END accompanying SLE versus those who only had SLE and subsequent observation, but there was no significant benefit in terms of DSS or recurrence-free survival [21]. This interesting outcome may be attributable to comorbidities or unhealthy lifestyle factors leading to only fitter patients receiving END, and the study advised considering END for clinically node-negative necks.

In our study, OND was present in only 7% of pathological specimens and 0.5% of the analyzed lymph nodes, which is within the range described in the literature. Advanced recurrent T-stage (rT3/rT4) was the only predictor for subclinical disease, which is consistent with previous reports [18]. We did not find tumor location to be a risk factor for OND, as has been suggested by other authors [3,18]. Of note, the surgical and observation groups had comparable oncologic outcomes in terms of RRFS and DSS. Thus, microscopic nodal disease, though present in a small percentage of patients, had limited overall impact and was unlikely the governing factor of the overall survival trends in our cohort. Along these lines, Gross et al. found that even though patients with supraglottic recurrence or advanced T classification tumors had a higher OND rate in their study, survival was not associated with END. Moreover, patients with recurrent hypopharyngeal subsites were at a higher risk for distant recurrence and death. Thus, the study concluded that survival outcomes likely hinged on underlying disease pathology, not surgical management [18].

Hilly et al. reported that END improved disease-free and overall survival in patients with locally advanced recurrent laryngeal squamous cell carcinoma (rT3 or rT4) and hypothesized that most series fail to show this advantage due to lack of stratification [22]. Our data do not support these findings as END did not confer significant survival advantages, even when properly stratifying by disease stage. However, considering the higher incidence of OND in patients with advanced stage (and the limited number of patients in most series), it is plausible to hypothesize that elective management of the neck might confer a survival advantage in this subset of patients.

Surgery in a radiated field carries an increased risk of postoperative wound complications [23,24]. Most authors agree that a modified radical neck dissection (levels I-V) is not warranted in such a setting, and a selective (lateral) neck dissection of levels II-IV is adequate according to the nodal drainage patterns described by Shah and Anderson [25]. Given the higher morbidity profile associated with formal lateral neck dissection in the salvage setting, it is certainly tempting to limit dissection to high-risk nodal basins. Indeed, several series suggest that a selective neck dissection of levels II-III, or even IIA and III, may be adequate for supraglottic carcinoma [5,26]. However, our findings do not support this approach, as microscopic nodal disease was found in all three levels of the lateral neck specimens. As such, we suggest that a formal lateral neck dissection should remain the procedure of choice for patients undergoing SLE. 

Surgical manipulation of the neck may cause fibrosis or disruption of the vascular supply in the radiated neck, potentially resulting in impaired wound healing. In patients who undergo a primary pharyngeal closure without regional or free tissue transfer, END has been correlated with an increased risk of PC fistula in the post-radiation setting [27]. However, most series fail to establish this association, as the risk of disruption of the pharyngeal closure is likely multifactorial and not directly related to the neck. Our study found that PC fistula was more common in the END group, but this difference was not statistically significant, even when accounting for the type of reconstruction performed (*p*=0.186). 

The impact of PET/CT was not thoroughly evaluated in our study. However, of the four patients with OND, only one had a preoperative PET/CT scan, which was negative for nodal disease (standardized uptake value <2). While this test has since become part of our preoperative workup, it is important to point out its relative lack of sensitivity (50%) for detecting OND [28]. Conversely, PET/CT is a valuable tool to assess clinically node-positive patients in the post-radiation setting, where it has demonstrated utility in preventing unnecessary surgical explorations [29]. Correlating with our pathological findings, we hypothesize that only one of the node-positive necks could have been identified preoperatively with a PET/CT scan as this neck had a 10 mm node. All remaining disease-positive lymph nodes were sub-centimeter and unlikely to be detected with this modality.

While this study provides valuable insights, it is crucial to acknowledge its limitations, mostly related to its retrospective design and limited access to certain data points and quality records. Since most of these patients were primarily treated in the community, data regarding the initial clinical and radiological status of the neck at presentation were inconsistent and hence not included in the analysis. Similarly, it was difficult to account for the significant variability in terms of organ preservation approach, radiation techniques, and fractionation schemes. Also, the study's single-center design may limit the generalizability of the findings, as results may be influenced by institution-specific practices or patient populations.

Though this study's two cohorts were very comparable in most aspects, surgical selection bias may always play a role as a confounding factor in a study of this nature. Moreover, the rate of chemoradiotherapy was higher in the END group, conceivably lowering the OND burden and increasing survival rates in this group. We believe such limitations reflect the challenges and complexities intrinsic to the management of patients with recurrent cancer. Whether END is “right” for a cN0 neck is still not an easy question to answer. Elective management of the neck must still be a shared decision with the individual patient - a decision weighing patient morbidity, lifestyle, frailty, age, and those factors commonly believed to put a patient at risk for OND. Moving forward, there is a critical need for future studies to enhance the quality of evidence in this field. This can be achieved through prospective studies with larger sample sizes to improve statistical power. Despite this study’s limitations, its findings should be a valuable addition to the growing body of evidence on the subject.

## Conclusions

In cN0 patients undergoing SLE, OND was present in only 7% of specimens, but positive lymph nodes were located in levels II, III, and IV. Given this indiscriminate distribution of OND, formal lateral neck dissection should be the preferred type of END to accompany SLE. An advanced recurrent stage (rT3-rT4) was the only predictor of OND in this study. END was not associated with a higher morbidity profile versus conservative management of the neck, yet the procedure conferred no measurable advantage in terms of disease control or survival, even when stratifying by tumor stage. As both groups had comparable oncologic outcomes in terms of RRFS and DSS, it is unlikely that OND had a significant impact on this cohort's overall survival trends.
